# Pterional craniotomy for occlusion of a basal temporal arteriovenous fistula

**DOI:** 10.3171/2025.7.FOCVID2587

**Published:** 2025-10-01

**Authors:** Anna L. Huguenard, Charuta G. Furey, Michael T. Lawton

**Affiliations:** Department of Neurosurgery, Barrow Neurological Institute, St. Joseph’s Hospital and Medical Center, Phoenix, Arizona

## Abstract

Atypical arteriovenous fistulas (AVFs) lack the parenchymal nidus observed in arteriovenous malformations (AVMs) and are not dural-based lesions supplied by meningeal arteries, unlike true dural AVFs. Atypical AVFs are parenchymally based with a nondural arterial supply. This video presents a man in his 50s with a remote carotid takedown for flow reduction of a described temporal AVM. Imaging showed a basal temporal AVF supplied by middle cerebral artery branches, coalescence of arterial feeders onto a large venous varix, and no intervening nidus. Dearterialization of the venous varix eliminated the shunting lesion. Atypical AVFs are often misdiagnosed but can be cured with microsurgical interruption.

The video can be found here: https://stream.cadmore.media/r10.3171/2025.7.FOCVID2587

## Transcript

This video will demonstrate a pterional craniotomy for occlusion of a basal temporal arteriovenous fistula.

### 0:28 Clinical Presentation.

The patient was in his early 50s. He presented for follow-up for a known left temporal AVM. He underwent multiple treatments related to this AVM, including a left carotid artery occlusion to reduce flow through the AVM, and clipping of a de novo right ophthalmic artery aneurysm. His neurological examination was intact.

### 0:52 Preoperative Imaging.

Here is his MRI, which shows this unusual pathology on the floor of the middle fossa, on the left side, underneath the temporal lobe. There is a large, partially thrombosed venous varix that is seen anteriorly coursing back toward the transverse sigmoid junction along a basal temporal vein. Notice the dilated middle cerebral artery in the sylvian fissure, which is enlarged due to this arteriovenous fistula drawing extra shunt flow.

### 1:27 Angiography.

His angiogram demonstrates a large, dilated middle cerebral artery trunk that emerges from the sylvian fissure, courses along the lateral temporal surface, and then winds underneath the basal temporal surface to arrive at the arteriovenous fistula. The artery appears to break up into other branches before diving into the venous varix.^1^ The lumen of the vein is smaller than what we saw on the MRI, indicating that part of the lumen is thrombosed.

### 2:01 Surgical Strategy and Patient Positioning.

A left pterional craniotomy was planned, and the exposure is shown here. Surgical strategy consisted of the left pterional craniotomy, dissection along the large middle cerebral artery afferent, identification of the feeding middle cerebral artery branches, IC-green videoangiography to confirm the site of the AV shunting, clip occlusion and division of two main feeding arteries, cauterization and division of the remaining middle cerebral artery feeding arteries, and, finally, circumdissection of the venous pouch.

### 2:39 Surgical Exposure.

Here’s an overview of the surgical exposure. We’re viewing the temporal lobe with the frontal lobe to the right, temporal lobe to the left, and the pterion at the top of the screen. Here you can see that dilated middle cerebral artery winding its way along the lateral temporal convexity. It makes a sharp turn underneath the temporal lobe and along the basal temporal surface.

### 3:03 Feeding Arteries.

Here multiple branches coalesce to a large trunk that dives into the venous varix underneath the temporal lobe. Here we see four such feeding arteries from the middle cerebral artery. This one here is cauterized and divided. Additional dissection around the varix gives you a better appreciation of how large this venous varix is.

### 3:38 Videoangiography.

Next, we do an IC-green videoangiogram here to confirm that these middle cerebral vessels are feeding into that common trunk before diving into the varix.^2^

### 3:49 Clip Occlusion.

Now you can see the two largest of the trunks. And I’m going to clip occlude both of these. Here our first clip goes on the smaller of the two branches. It can now be cauterized and divided. Next, the second clip goes on the larger of the two arteries. It too can be cauterized and divided.

### 4:25 Artery Cauterization.

Now some smaller branches, more anteriorly on the basal surface, are divided. As these arteries are cut, we get a better appreciation of the full extent of this varix. Here’s that fourth artery joining or coalescing with the trunk, and this too is cauterized and divided. Now all four of these main trunks have been disconnected from this arterial trunk along the basal surface. That arterial trunk is now dearterialized and can be cauterized and divided. Some additional trunks were found more medial to this other trunk. Here the main trunk is divided.

### 5:22 Venous Varix Disconnection.

With that, we can now follow the trunk all the way to its union with the venous varix. Here you can see some additional small feeders. And now the feeding arteries have been essentially disconnected. Now I’m going around the varix just making sure that there aren’t other additional feeders to the varix. The small ones are seen there, and these are all divided. The varix is now in full view. It was once pulsatile. Now it is quiet, and I’m doing some additional dissection around the varix to make sure that all feeders to it have been occluded. Here’s our final overview, showing a very quiet venous varix with no longer any arterial input into it.

### 6:08 Postoperative Outcome and Imaging.

The patient tolerated the procedure well. He had no new neurological deficits and was discharged home. His postoperative angiogram showed no further arteriovenous shunting and no filling of that dilated venous varix on the middle cranial fossa floor.

### 6:26 Conclusions.

In conclusion, this case demonstrates an unusual basal temporal arteriovenous fistula that was not truly a parenchymal AVM or a dural AV fistula, but something in between.^3–5^ Intraoperatively, this lesion had multiple enlarged feeding arteries on the basal temporal surface that joined a dilated and partially thrombosed varix on the middle fossa floor, without intervening intraparenchymal nidus. Microsurgical clip ligation and/or coagulation of arterial afferents effectively dearterialized this lesion and eliminated the shunt. The size and location of the varix precluded occlusion and resection of the efferent vein.^6^ Thank you.

## Acknowledgments

We thank the staff of Neuroscience Publications at Barrow Neurological Institute for assistance with manuscript preparation.

## Disclosures

Dr. Huguenard reported stock ownership in and serving as a board member for Aurenar, outside the submitted work.

## Author Contributions

Primary surgeon: Lawton. Assistant surgeon: Huguenard, Furey. Editing and drafting the video and abstract: all authors. Critically revising the work: Lawton, Huguenard. Reviewed submitted version of the work: all authors. Approved the final version of the work on behalf of all authors: Lawton. Supervision: Lawton.

## Supplemental Information

### Patient Informed Consent

The necessary patient informed consent was obtained in this study.
